# Assessment of Public Stigma Towards People with Mental Health Problems

**DOI:** 10.3390/nursrep16040126

**Published:** 2026-04-09

**Authors:** Lorena Liñán-Díaz, María Desamparados Bernat-Adell, Núria Vives-Díaz, Vicente Bernalte-Martí

**Affiliations:** Nursing Department, Faculty of Health Sciences, Jaume I University, 12071 Castellón de la Plana, Spain; al430912@uji.es (L.L.-D.); al414124@uji.es (N.V.-D.); bernalte@uji.es (V.B.-M.)

**Keywords:** attitude to mental health, community participation, mental disorders, nursing, social stigma, socioeconomic factors, social rejection

## Abstract

**Background/Objectives**: This study aimed to assess public stigma toward people with mental health problems and to examine the association between stigma and socioeconomic characteristics, personal mental health history, and contact with individuals with mental health problems. **Methods**: This observational, descriptive, and cross-sectional study was conducted among the general population in Spain using a sample of 404 participants, the majority of whom were women (71%), with a median age of 38 years (IQR = 26–49); most participants (86.4%) lived in urban areas. The participants completed a self-administered online questionnaire that explored socioeconomic variables and the Community Attitudes towards Mental Illness Scale (CAMI-S, Spanish version). Non-parametric tests (Mann–Whitney U, Kruskal–Wallis, and Spearman correlation), multiple linear regression, and statistical power analyses were performed. **Results**: The mean CAMI-S total score was 84.89 (SD = 11.122) out of 100, indicating relatively favourable attitudes toward people with mental health problems. Statistically significant associations (*p*-value ≤ 0.05) were found between CAMI-S scores and variables such as gender, age, place of residence, educational level, mental health disorder, and close contact with someone with mental health disorders. The regression model revealed four significant predictors of lower stigma: identifying as female (β = 2.523; *p* = 0.037), having a medium or higher educational level (β = 5.061; *p* = 0.002), experiencing a mental health diagnosis (β = 4.535; *p* = 0.014), and close contact (β = 4.183; *p* < 0.001). **Conclusions:** Social stigma toward people with mental health problems in Spain appears to be generally low, reflecting positive attitudes toward community integration. Being female, having higher education, and personal or close contact with mental health problems are associated with lower stigma.

## 1. Introduction

Mental disorders are defined in the Diagnostic and Statistical Manual of Mental Disorders (DSM-V-TR) as a “syndrome characterized by a clinically significant disturbance in an individual’s cognition, emotional regulation, or behaviour, reflecting a dysfunction in the psychological, biological, or developmental processes underlying mental functioning” [1].

The concept of “mental disorder” is closely associated with the term “stigma”, a concept that comes from Ancient Greece, as a reference to physical marks placed on individuals to indicate negative or unusual moral status. These marks, often visible, served as a warning sign that the bearer was considered dishonourable and should be avoided, especially in public spaces. In 1963, sociologist Erving Goffman reintroduced the concept into the social sciences, defining stigma as an attribute that discredits an individual and reduces them to a lesser status in the perception of others [2].

Building on this initial definition, Link and Phelan proposed a structural approach to stigma [3], which was later revised and simplified by Andersen, Varga, and Folker. This group identified four key elements in the construction of stigma: labelling, negative stereotypes, linguistic separation, and power asymmetry. In their revision, they omitted two components of the original definition due to redundancy: status loss and discrimination, and emotional reaction. This approach enables an understanding of stigma as a collective social construct affecting groups, though its consequences are experienced individually [4,5].

Regarding typology, the classification proposed by Pryor and Reeder (2011) [6] remains a key reference to understanding the different forms of stigma: social stigma, self-stigma, associative stigma, and structural stigma. This framework helps to analyse how stigma operates in different contexts, and the following types of stigma were identified [6]:

- Public or social stigma: Refers to society’s negative attitudes and discriminatory behaviours toward people with mental health problems.
- Self-stigma: Involves the internalization of those attitudes, where individuals adopt society’s negative perception of their own condition.
- Structural stigma: Includes the policies, laws, and institutional practices that perpetuate inequality and restrict rights.
- Associative stigma: Involves prejudice and discrimination toward people connected with someone who is stigmatized, such as relatives or friends.

This study focuses on social stigma, which comprises three interdependent dimensions:

1. Stereotypes (cognitive): Socially held beliefs about mental illness (e.g., danger, weakness, incompetence).
2. Prejudice (emotional): Negative emotional reactions toward the stigmatized group.
3. Discrimination (behavioural): Resulting actions, such as social distancing or unequal treatment [7,8].

This tripartite model has also been systematized in the Mental Illness Stigma Framework, which conceptualizes stigma as a dynamic process involving these three interconnected components [9].

Social stigma negatively impacts individuals, reducing self-esteem, increasing feelings of guilt, and leading to social isolation. It also limits opportunities in education, employment, and community participation. The fear of social rejection may become a barrier to seeking professional help [10,11]. Because of this, stigma is often described as a “second illness,” obstructing prevention, treatment, and recovery [12].

Discrimination against people with mental health takes many forms, including social exclusion in education, the workplace, and community life, as well as loss of property, inheritance, and poorer healthcare compared to physical conditions. These pervasive and persistent barriers negatively affect individuals, families, communities, and society across all cultures and undermine fundamental human rights that are meant to be equally guaranteed to all people [13].

Stigma also affects healthcare professionals and, significantly, nurses who work in the mental health field, as their daily mission is not only to care for people with mental health problems but also to minimize the social stigma and social discrimination that accompany these people [14]. The term “mental disorder” is used in quotation marks in this study to emphasize that, although it is widely used in the scientific literature and in diagnostic manuals such as the DSM-V-TR and the International Classification of Diseases (ICD-11), it can function as a diagnostic label that reinforces associated stigma. Therefore, whenever possible, the term “mental health problem” will be used, with the aim of promoting a more respectful and person-centered approach [15].

Although several studies have explored public stigma toward mental illness, evidence from the Spanish general population remains limited, particularly regarding the influence of sociodemographic characteristics and personal contact with individuals experiencing mental health problems.

Therefore, this study aimed to assess public stigma toward people with mental health problems in the Spanish general population and to examine whether sociodemographic characteristics, personal mental health history, and close contact with individuals with mental illness are associated with stigma levels.

## 2. Materials and Methods

### 2.1. Study Design, Participants, and Setting

The study follows an observational, descriptive, and cross-sectional design. It was conducted between September 2023 and April 2025. It was carried out among the general population in Spain, involving individuals who voluntarily participated after being informed of the research objectives.

The target population consisted of individuals who met the established inclusion criteria. The sample size was calculated assuming that the total number of adults living in Spain who met the inclusion criteria was unknown, and the population was assumed to be infinite (N = ∞). A 95% confidence level (Z = 1.96), 5% precision (d = 0.05), and an expected proportion of 50% (*p* = 0.5) were used. Based on these parameters, the minimum sample size was 384 participants. Due to the exploratory nature of the study, a non-probabilistic convenience sampling strategy was used to collect the sample. Participants were recruited through social media platforms such as Instagram, WhatsApp, and Twitter. Ultimately, 404 individuals were collected throughout the sampling period.

### 2.2. Inclusion and Exclusion Criteria

Participants eligible for inclusion in the study were required to be over 18 years of age, reside in Spain, and be able to read and comprehend Spanish. Individuals who had obtained a university degree in nursing, medicine, or psychology were excluded from participation because their training could influence their attitudes toward mental illness and potentially bias the estimation of stigma in the general population.

### 2.3. Study Variables

On the one hand, the following variables were defined as independent: the socioeconomic variables—gender, age, place of residence, marital status, children, educational level, employment status, having a “mental disorder”, and having close contact with a family member, friend, or acquaintance with mental health problems. On the other hand, the main dependent variable was the total score of the CAMI-S scale. Additionally, the 20 items from the Spanish version of the CAMI-S scale (Community Attitudes towards Mental Illness, Swedish version) were analysed in the bivariate analysis, and they are expressed by a 5-point Likert scale response format (1 = strongly disagree; 2 = somewhat disagree; 3 = neither agree nor disagree; 4 = somewhat agree; 5 = strongly agree). Finally, the total score of the CAMI-S scale was calculated by recoding the negatively phrased items and generating a quantitative variable, which was named “total”.

### 2.4. Measurement Instruments

In 1980, Taylor and Dear developed the CAMI scale (Community Attitudes towards the Mentally Ill) as a tool to predict and explain society’s perception of people with severe mental health problems [16]. This version includes 40 questions grouped into four factors: authoritarianism, benevolence, social restrictiveness, and community mental health ideology, with satisfactory validity and reliability results. Later, Högberg et al. removed items with poor correlation, reducing the scale to 20 items, known as CAMI-S. The total questionnaire score ranges from 20 to 100, where higher scores reflect more suitable attitudes toward integration. Three factors are also examined: integration and contact, social distance, and dangerousness and avoidance [17].

In this study, the Spanish adapted and validated version of the CAMI-S scale by Sastre-Rus et al. [17] was used to assess social stigma toward people with mental health problems. Data were collected via a self-administered questionnaire by the platform Qualtrics XM (XM Platform), distributed between February and June 2024 through Instagram, Twitter, and the instant messaging application WhatsApp.

### 2.5. Ethical Considerations

The study adhered to the Declaration of Helsinki (Declaration of Helsinki of the WMA—Ethical Principles for Medical Research Involving Human Participants, 2024) for research involving human subjects. The project was approved by the Human Research Ethics Committee (CEISH) of the University Jaume I under code CEISH/134/2023.

### 2.6. Data Analysis

To assess the internal reliability of the questionnaire, variables were recoded, obtaining a Cronbach’s alpha coefficient of α = 0.860, indicating that the CAMI-S scale is reliable and suitable for measuring social stigma in the sample analysed. For descriptive analysis, frequencies and percentages were evaluated for qualitative variables, and quantitative variables were expressed as the median, interquartile range (IQR), and minimum and maximum values. For the total CAMI-S score, the mean and standard deviation were used, given its normal distribution.

The Kolmogorov–Smirnov test revealed that the variables did not follow a normal distribution. Therefore, non-parametric tests were applied for bivariate analyses (Mann–Whitney U test, Spearman’s correlation coefficient, and the Kruskal–Wallis’s test)

The software G*Power 3.1 was used to calculate the effect size (Cohen’s d) and estimate the statistical power (1 − β) for comparisons that yielded statistically significant results. Effect sizes were interpreted following Cohen’s (1988) recommendations (Around 0.20: small effect, around 0.50: medium effect, above 0.80: large effect) [18].

Lastly, a multiple linear regression analysis was conducted using the backward stepwise method to identify the socioeconomic variables that best predict the total CAMI-S score. Independent variables were transformed into dummy variables (0 or 1, based on specific categories) using the highest-risk category as the reference group. The model’s goodness of fit was assessed using the adjusted coefficient of determination (Adjusted R^2^). The statistical analysis was carried out using IBM SPSS Statistics (version 29). A significance level of *p* ≤ 0.05 was applied in all cases.

## 3. Results

The study included a sample of 404 participants, of which 71% (n = 287) identified as women, and 29% (n = 117) identified as men. No participant identified as non-binary. The median age was 38 years (IQR 49–26), with a range from 18 to 78 years. Additionally, most participants (86.4%, n = 349) lived in urban areas.

Regarding marital status, the 42.8% (n = 173) were married, 28.7% (n = 116) were in a relationship, 28% (n = 113) were single, and the 0.5% (n = 2) were widowed. In relation to parenthood, 50.5% (n = 204) reported not having children. In terms of education, the 55.9% (n = 226) had university studies, 31.7% (n = 128) had secondary education, 11.4% (n = 46) had primary education, and a 1% (n = 4) had no formal education.

As for employment status, the majority were economically active (75.2%, n = 304). Furthermore, 90.6% (n = 366) of participants had never experienced mental health problems, while 9.4% (n = 38) reported having a mental health diagnosis. In addition, 53.2% (n = 215) had no close contact with people with mental health problems, while 46.8% (n = 189) did.

This socioeconomic profile provides the basis for examining how different characteristics of the population may be associated with variations in public stigma toward mental health problems.

The mean total score of the CAMI-S scale was 84.89 ± 11.122. The results of each item from the CAMI-S questionnaire are presented as tables in the Supplemental Material (Tables S1–S3).

The following tables present the bivariate analysis examining the relationship between socioeconomic variables and the items of the CAMI-S scale. Effect size and statistical power were calculated only for those variables that demonstrated prior statistical significance. These results are described after each table, focusing exclusively on those with medium to high relevance. The items are grouped according to the factors established by Sastre-Rus et al. [17].

Table 1 presents the results for factor 1 (Integration and Contact) based on gender, age, place of residence, and marital status.

The most relevant findings regarding effect size and statistical power analyses were observed for Item 7, “Mental illness is an illness like any other,” where younger participants showed significantly stronger agreement, with a medium effect size (d = 0.460) and very high statistical power (1 − β = 1.000).

Similarly, Item 16, “The best therapy for many people with mental disorders is to be part of the community,” was significantly associated with marital status. This result also demonstrated a medium effect size (d = 0.439) and high statistical power (1 − β = 0.999). The findings suggest that being married is associated with greater acceptance and lower levels of social stigma toward individuals with mental health problems.

Table 2 presents the results for factor 1 (Integration and Contact) based on the socioeconomic variables: educational level, employment status, mental disorder, and close contact.

In this case, the most relevant findings regarding effect size and statistical power analyses were also observed for Item 7, “Mental illness is an illness like any other.” Individuals who were actively employed showed significantly stronger agreement, with a medium effect size (d = 0.464) and very high statistical power (1 − β = 1.000).

Similarly, Item 12, “It is frightening to think that some people with mental disorders live in residential neighbourhoods,” was significantly associated with employment status. This result also demonstrated a medium effect size (d = 0.530) and high statistical power (1 − β = 1.000). These findings suggest that individuals who are employed tend to feel less frightened about living near people with mental health problems.

Table 3 presents the results of the analysis of each item included in the CAMI-S scale factor related to social distance. Only statistically significant associations with socioeconomic variables that may influence these items are reported.

Regarding the social distance factor, the most meaningful findings in terms of effect size and statistical power were observed for Item 13, “The best way to treat people with a mental disorder is through hospitalization.” Individuals with a higher level of education showed stronger disagreement with this statement, with a medium effect size (d = 0.417) and very high statistical power (1 − β = 0.999).

Similarly, Item 14, “Neighbours have nothing to fear from people coming into their neighbourhood to use mental health services,” was also associated with employment status. This result demonstrated a medium effect size (d = 0.409) and high statistical power (1 − β = 0.999).

The results of analyzing factor 3 (Dangerousness and Avoidance) based on socioeconomic factors are presented in Table 4.

Regarding the Dangerousness and Avoidance factor, the most relevant findings in terms of effect size and statistical power were observed for Item 20, “People with mental disorders should be isolated from the rest of the community.” Individuals with a higher level of education showed stronger disagreement with this statement, with a medium effect size (d = 0.589) and very high statistical power (1 − β = 1.000). Similarly, individuals who reported close contact with people with mental disorders also showed strong disagreement, with a medium effect size (d = 0.453) and very high statistical power (1 − β = 1.000).

On the other hand, participants with children showed a greater tendency to agree with the use of isolation measures. This result also demonstrated a medium effect size (d = 0.469) and high statistical power (1 − β = 1.000).

The final multiple linear regression model identified four significant predictors of the total variable representing community integration: gender, educational level, having a “mental disorder”, and having close contact. The adjusted coefficient of determination (Adjusted R^2^) was 0.109, indicating that 10.9% of the variance in the dependent variable is explained by the variables included in the model. The results obtained were as follows (Table 5).

## 4. Discussion

The findings of this study indicate an overall favorable attitude toward the community integration of people with mental health problems, as reflected by the mean CAMI-S score (84.89). This result is consistent with previous studies conducted in similar populations, such as medical residents, where comparable scores have been reported [19,20]. These findings suggest a general trend toward reduced stigma in the population.

Despite this overall positive trend, the results highlight the influence of several socioeconomic factors, which reveal a more complex and nuanced pattern of stigma. In particular, factors such as higher educational level, personal experience with mental health problems, and close contact with affected individuals were consistently associated with more favorable attitudes. These findings are in line with previous research suggesting that familiarity and knowledge play a key role in reducing stigma [21,22,23]. In addition, Ruiz et al. reported that students in health-related disciplines exhibit lower levels of stigma compared to those in other fields [24]. Similarly, previous studies have reported that individuals with lower educational levels tend to show more stigmatizing attitudes [21,25].

Similarly, gender differences were observed, with women showing lower levels of stigmatization and greater willingness for community integration, consistent with findings from Schroeder and Dey [26,27]. However, contradictory findings in other cultural contexts [21] suggest that these differences may be influenced by sociocultural and educational factors rather than gender alone.

In contrast, certain variables were associated with more ambivalent or less favorable attitudes. For example, although younger participants exhibited lower levels of stigma, they also reported a higher perception of risk. This apparent contradiction has been described in previous literature [22,28] and may reflect increased awareness of mental health issues without a parallel reduction in perceived dangerousness.

Likewise, the results related to place of residence differed from previous findings. While earlier studies have associated rural environments with higher levels of stigma due to limited access to information and resources [26,29], our results indicate greater social rejection among individuals living in urban areas. This discrepancy may be influenced by contextual factors or characteristics of the sample.

Other variables, such as marital status, employment, and parenthood, showed heterogeneous results. Married individuals expressed more favorable attitudes toward community inclusion, whereas parenthood was associated with increased perceptions of dangerousness and avoidance. This finding may be related to protective concerns, as previous studies have shown that parents tend to perceive mental health symptoms as more severe and adopt more critical attitudes [30]. However, previous studies have reported inconsistent results regarding parenthood [31]. In addition, employment status has shown mixed associations with stigma, with Alonso et al. finding lower stigma among economically active individuals [25], whereas Rita et al. reported more empathetic attitudes in non-working groups such as housewives [23].

Furthermore, although contact with people with mental health problems was associated with lower levels of stigma, it should be noted that contact alone may not be sufficient to eliminate stigmatizing attitudes. Previous research has shown that familiarity is linked to lower stigma and greater benevolence [22,23], although even mental health professionals may maintain certain stereotypes [32], suggesting that stigma is influenced by broader structural and cultural factors.

The statistical power of the analyses supports the robustness of these findings, as several associations were identified with adequate power (1 − β > 0.80). This reinforces the robustness of our findings, suggesting that the observed relationships are unlikely to be due to chance and are potentially replicable in similar contexts.

The final multiple linear regression model identified four significant predictors of the total variable representing a suitable community integration: gender, medium and higher educational level, suffering a mental health problem, and having close contact. The adjusted coefficient of determination (Adjusted R^2^) was 0.109, indicating that 10.9% of the variance in the dependent variable is explained by the variables included in the model. This suggests that, although the identified predictors are relevant, most of the variation in the outcome may be influenced by other factors not included in the model or may be related to the sampling method. However, our results can help to better understand people’s behavior in the face of mental health problems.

Overall, these results highlight the complexity of stigma toward people with mental health problems and reinforce the need to consider multiple interacting factors when designing interventions aimed at its reduction.

## 5. Limitations

The authors acknowledge that the study has certain limitations. Firstly, the use of non-probabilistic convenience sampling limits the ability to generalize the results to the entire Spanish adult population. Consequently, the findings may not be representative and should be interpreted with caution beyond the specific group of respondents reached through the platforms used to collect the information.

In addition, the online data collection may have introduced self-selection bias, as it is likely that only people with access to social media were included, therefore excluding less connected segments of the population.

## 6. Conclusions

An overall favourable trend toward the community integration of individuals with mental health problems is observed, although relevant differences persist according to various socioeconomic variables. In this regard, women exhibit lower levels of stigmatization, which translates into a greater willingness to support social inclusion. Likewise, married individuals show a stronger tendency to endorse the community participation of this population.

Age also emerges as an influential factor, as older individuals tend to display higher levels of stigma, whereas younger individuals, although generally more inclusive in their attitudes, perceive a greater level of risk. Similarly, not having children is associated with less prejudiced attitudes, particularly in relation to dangerousness and avoidance. In contrast, individuals with children appear to adopt more cautious positions.

Finally, higher educational attainment is consistently associated with lower levels of stigmatization and greater support for social inclusion. This pattern is also observed among individuals living in rural or semi-rural areas, as well as those who are economically active. Moreover, both personal experience of having a mental disorder and close contact with individuals affected by mental health conditions contribute to the development of more positive and empathetic attitudes toward this group.

## 7. Future Research

Future nursing research should continue exploring and analyzing the phenomenon of social stigma that affects people with mental health problems to generate more and better results that support health programs and scientific evidence.

Quantitative and especially qualitative studies can help to explore patients’ lived experiences, as well as healthcare professionals’ attitudes and beliefs.

The authors will carry on systematically collecting data through surveys and interviews that explore negative or positive attitudes towards mental health problems.

## Figures and Tables

**Table 1:** Statistical results for items related to Factor 1 (Integration and Contact).

Item	Gender	Age	Place of Residence	Marital Status
	Value (*U*) (*p*-Value)	Value (*r*) (*p*-Value)	Value (*K*) (*p*-Value)	Value (*K*) (*p*-Value)
2	**19,648.5** (0.006) *	−0.005 (0.915)	0.398 (0.820)	2.649 (0.449)
7	18,287.5 (0.123)	**0.212** (<0.001) **	**8.475** (0.014) *	3.662 (0.3)
8	18,058.5 (0.129)	**0.109** (0.028) *	**8.949** (0.011) *	0.323 (0.956)
12	15,532.5 (0.132)	0.006 (0.898)	2.314 (0.314)	2.137 (0.544)
15	17,048 (0.803)	0.067 (0.182)	2.168 (0.338)	1.626 (0.654)
16	**18,884** (0.02) *	0.060 (0.226)	3.992 (0.136)	**9.236** (0.026) *
17	17,913.5 (0.085)	0.043 (0.392)	0.837 (0.658)	3.596 (0.308)
18	**18,717.5** (0.013) *	0.094 (0.059)	1.644 (0.440)	3.913 (0.271)
19	18,002.5 (0.065)	0.031 (0.540)	2.571 (0.277)	2.972 (0.396)

Note. Bold values indicate statistically significant results. Mann–Whitney U test (*U*); Spearman’s correlation coefficient (*r*); Kruskal–Wallis test (*K*); Effect size (Cohen’s d); *p*-value ≤ 0.05 *; *p*-value < 0.001 **.

**Table 2:** Statistical results for items related to Factor 1 (Integration and Contact).

Item	Educational Level	Employment Status	“Mental Disorder”	Close Contact
	Value (*K*) (*p*-Value)	Value (*K*) (*p*-Value)	Value (*U*) (*p*-Value)	Value (*U*) (*p*-Value)
2	**32.766** (<0.001) **	5.980 (0.113)	**4573.5** (<0.001) **	**15,020** (<0.001) **
7	5.212 (0.157)	**17.265** (<0.001) **	6059 (0.152)	18,413.5 (0.075)
8	**23.732** (<0.001) **	6.271 (0.099)	**5644** (0.015) *	19,162 (0.209)
12	**19.84** (<0.001) **	**15.439** (0.001) **	**8009** (0.05) *	**23,087** (0.003) *
15	**8.973** (0.03) *	3.380 (0.337)	6170 (0.239)	**17,618** (0.018) *
16	6.940 (0.074)	6.417 (0.093)	6171 (0.177)	**16,452.5** (<0.001) **
17	**9.439** (0.024) *	6.402 (0.094)	6637.5 (0.451)	**18,358** (0.006) *
18	**14.847** (0.002) *	4.043 (0.257)	**5622** (0.007) *	**16,863** (<0.001) **
19	3.003 (0.391)	1.697 (0.638)	6195 (0.073)	19,522.5 (0.272)

Note. Bold values indicate statistically significant results.Mann–Whitney U test (U); Spearman’s correlation coefficient (r); Kruskal–Wallis test (K); *p*-value ≤ 0.05 *; *p*-value < 0.001 **.

**Table 3:** Statistical results of items related to Factor 2 (Social Distance).

Item	Gender	Educational Level	Employment Status	“Mental Disorder”	Close Contact
	Value (*U*) (*p*-Value)	Value (*K*) (*p*-Value)	Value (*K*) (*p*-Value)	Value (*U*) (*p*-Value)	Value (*U*) (*p*-Value)
1	18,100.5 (0.12)	4.649 (0.199)	0.955 (0.812)	6277 (0.212)	19,100.5 (0.189)
3	18,483 (0.064)	**16.914** (<0.001) **	1.406 (0.704)	**5560** (0.018) *	**18,273** (0.042) *
4	15,440 (0.154)	**9.983** (0.019) *	5.735 (0.125)	**8928.5** (0.001) *	**25,093.5** (<0.001) **
5	**14,069.5** (0.008) *	**13.633** (0.003) *	6.548 (0.088)	**8896.5** (0.003) *	**24,563.5** (<0.001) **
6	14,989 (0.075)	**16.773** (<0.001) **	4.147 (0.246)	8136.5 (0.069)	**24,369** (<0.001) **
13	**13,558.5** (0.001) *	**24.387** (<0.001) **	**16.179** (0.001) *	**8334** (0.029) *	**25,150.5** (<0.001) **
14	**19,352.5** (0.007) *	**17.411** (<0.001) **	2.697 (0.441)	**5312.5** (0.007) *	**17,206** (0.003) *

Note. Bold values indicate statistically significant results. Mann–Whitney U test (U); Spearman’s correlation coefficient (r); Kruskal–Wallis test (K); *p*-value ≤ 0.05 *; *p*-value < 0.001 **.

**Table 4:** Statistical results of items related to Factor 3 (Dangerousness and Avoidance).

Item	Gender	Age	Children	Educational Level	Employment Status	“Mental Disorder”	Close Contact
	Value (*U*) (*p*-Value)	Value (*r*) (*p*-Value)	Value (*U*) (*p*-Value)	Value (*K*) (*p*-Value)	Value (*K*) (*p*-Value)	Value (*U*) (*p*-Value)	Value (*U*) (*p*-Value)
9	**19,607** (0.005) *	−0.013 (0.792)	**22,873** (0.026) *	**23.186** (<0.001) **	6.807 (0.078)	5721.5 (0.057)	**16,023.5** (<0.001) **
10	15,438 (0.095)	0.072 (0.147)	**18,565.5** (0.04) *	**28.555** (<0.001) **	7.031 (0.071)	7934.5 (0.06)	**22,739** (0.007) *
11	**14,433.5** (0.018) *	**−0.097** (0.05) *	19,447.5 (0.385)	6.262 (0.1)	2.201 (0.532)	**8674** (0.007) *	**23,681.5** (0.002) *
20	16,075.5 (0.337)	0.090 (0.071)	**18,660** (0.034)*	**27.667** (<0.001) **	**14.154** (0.003) *	**8121** (0.015) *	**22,695.5** (0.004) *

Note. Bold values indicate statistically significant results. Mann–Whitney U test (U); Spearman’s correlation coefficient (r); Kruskal–Wallis test (K); *p*-value ≤ 0.05 *; *p*-value < 0.001 **.

**Table 5:** Unstandardized coefficients of the final model.

Variable	Coefficient (*β*)	Standard Error	*p*-Value
Constant (Total)	85.001	2.518	<0.001
Gender (Woman)	2.523	1.205	0.037
Educational level (Secondary and University)	5.061	1.604	0.002
“Mental disorder” (Yes)	4.535	1.831	0.014
Close contact (Yes)	4.183	1.123	<0.001

Note. Multiple linear regression, backward stepwise method.

## Data Availability

The original data presented in the study are openly available upon request from the authors.

## Supplementary Materials

The following supporting information can be downloaded at: https://www.mdpi.com/article/10.3390/nursrep16040126/s1, Table S1. CAMI-S scale items related to Factor 1 (integration and contact). Table S2. CAMI-S Items Related to Factor 2 (Social Distance). Table S3. CAMI-S Items Related to Factor 3 (Dangerousness and Avoidance).

## Public Involvement Statement

Participants were recruited through social media platforms such as Instagram, WhatsApp, and Twitter. Ultimately, 404 individuals were collected throughout the sampling period.

## Guidelines and Standards Statement

This manuscript was drafted against the STROBE (Strengthening the Reporting of Observational Studies in Epidemiology) guidelines.

## Abbreviations

The following abbreviations are used in this manuscript:

DSM-V-TR	Diagnostic and Statistical Manual of Mental Disorders, Fifth Edition, Text Revision
ICD-11	International Classification of Diseases, 11th Edition
CAMI-S	Community Attitudes towards Mental Illness
IBM SPSS	International Business Machines Sta
SPSS	Statistical Package for the Social Sciences
IQR	Interquartile range
CEISH	Ethics Committee for Research Involving Human Subjects
STROBE	Strengthening the Reporting of Observational Studies in Epidemiology
