# Supplementary material for: Assessment of Public Stigma Towards People with Mental Health Problems
Source: Nurs Rep. 2026 Apr 9;16(4):126. doi: 10.3390/nursrep16040126 (PMC13119059; doi:10.3390/nursrep16040126)
Supplement: Supplementary file 1 [file nursrep-16-00126-s001.zip › nursrep-4213593-supplementary.pdf]

Supplementary materials.

Table S1. *CAMI-S scale items related to Factor 1 (integration and contact).*

Item	Value	n[1]	%[2]
2. Most people who have experienced a mental disorder are capable of caring for children.	Strongly disagree	26	6.4
	Somewhat disagree	59	14.6
	Neither agree nor disagree	79	19.6
	Somewhat agree	124	30.7
	Strongly agree	116	28.7
7. Mental illness is an illness like any other.	Strongly disagree	30	7.3
	Somewhat disagree	60	14.9
	Neither agree nor disagree	16	4.0
	Somewhat agree	80	19.8
	Strongly agree	218	54.0
8. We need a more tolerant attitude toward people with mental disorders.	Strongly disagree	13	3.2
	Somewhat disagree	9	2.2
	Neither agree nor disagree	18	4.5
	Somewhat agree	72	17.8
	Strongly agree	292	72.3
12. It is frightening to think that some people with mental disorders live in residential neighbourhoods.	Strongly disagree	293	72.5
	Somewhat disagree	59	14.6
	Neither agree nor disagree	37	9.2
	Somewhat agree	8	2.0
	Strongly agree	7	1.7
15. Less emphasis should be placed on protecting people from the mentally ill.	Strongly disagree	74	18.3
	Somewhat disagree	39	9.6
	Neither agree nor disagree	92	22.8
	Somewhat agree	71	17.6
	Strongly agree	128	31.7
16. The best therapy for many people with mental disorders is to be part of the community.	Strongly disagree	7	1.7
	Somewhat disagree	5	1.2
	Neither agree nor disagree	35	8.7
	Somewhat agree	96	23.8
	Strongly agree	261	64.6

Item	Value	n[1]	%[2]
17. People with mental disorders should not be marginalized.	Strongly disagree	9	2.2
	Somewhat disagree	2	0.5
	Neither agree nor disagree	8	2.0
	Somewhat agree	40	9.9
	Strongly agree	345	85.4
18. Mental health services should, as far as possible, be integrated into community services.	Strongly disagree	5	1.2
	Somewhat disagree	2	0.5
	Neither agree nor disagree	19	4.7
	Somewhat agree	64	15.8
	Strongly agree	314	77.8
19. No one has the right to exclude people with mental disorders from their community.	Strongly disagree	4	1.0
	Somewhat disagree	8	2.0
	Neither agree nor disagree	11	2.7
	Somewhat agree	37	9.2
	Strongly agree	344	85.1

Note. [1] Absolute frequency; [2] Percentage

Table S2. *CAMI-S Items Related to Factor 2 (Social Distance)*

Item	Value	n[1]	%[2]
1. Citizens should accept the establishment of mental health centres in their neighbourhood for the benefit of the community.	Strongly disagree	33	8.1
	Somewhat disagree	4	1.0
	Neither agree nor disagree	21	5.2
	Somewhat agree	56	13.9
	Strongly agree	290	71.8
3. The presence of mental health services in residential neighbourhoods does not endanger the residents.	Strongly disagree	33	8.2
	Somewhat disagree	24	5.9
	Neither agree nor disagree	30	7.4
	Somewhat agree	60	14.9
	Strongly agree	257	63.6
4. Mental health centres should be located outside of residential neighbourhoods.	Strongly disagree	237	58.7
	Somewhat disagree	59	14.6
	Neither agree nor disagree	71	17.6
	Somewhat agree	26	6.4
	Strongly agree	11	2.7
5. Allowing people with mental disorders to live in residential neighbourhoods may help in their recovery but poses too many risks to neighbours.	Strongly disagree	160	39.7
	Somewhat disagree	87	21.5
	Neither agree nor disagree	81	20.0
	Somewhat agree	57	14.1
	Strongly agree	19	4.7
6. There are good reasons for citizens to oppose the location of mental health services in their neighbourhood.	Strongly disagree	176	43.6
	Somewhat disagree	94	23.3
	Neither agree nor disagree	59	14.6
	Somewhat agree	47	11.6
	Strongly agree	28	6.9
13. The best way to treat people with a mental disorder is through hospitalization.	Strongly disagree	203	50.3
	Somewhat disagree	109	27.0
	Neither agree nor disagree	53	13.1
	Somewhat agree	28	6.9
	Strongly agree	11	2.7
	Strongly disagree	8	2.0
	Somewhat disagree	33	8.1
	Neither agree nor disagree	36	8.9
	Somewhat agree	90	22.3

Item	Value	n[1]	%[2]
14. Neighbours have nothing to fear from people coming into their neighbourhood to use mental health services.	Strongly agree	237	58.7

Note. [1] Absolute frequency; [2] Percentage

Table S3. *CAMI-S Items Related to Factor 3 (Dangerousness and Avoidance)*

Item	Value	n[1]	%[2]
9. People with mental disorders are less dangerous than most people think.	Strongly disagree	17	3.2
	Somewhat disagree	26	2.2
	Neither agree nor disagree	18	4.5
	Somewhat agree	74	17.8
	Strongly agree	292	72.3
10. It is best to avoid people with mental disorders	Strongly disagree	302	74.8
	Somewhat disagree	63	15.6
	Neither agree nor disagree	27	6.7
	Somewhat agree	5	1.2
	Strongly agree	7	1.7
11. I would not like to have a neighbour who has had a mental disorder.	Strongly disagree	191	47.3
	Somewhat disagree	98	24.3
	Neither agree nor disagree	82	20.3
	Somewhat agree	24	5.9
	Strongly agree	9	2.2
20. People with mental disorders should be isolated from the rest of the community.	Strongly disagree	323	80.0
	Somewhat disagree	49	12.0
	Neither agree nor disagree	20	5.0
	Somewhat agree	6	1.5
	Strongly agree	6	1.5

Note. [1] Absolute frequency; [2] Percentage
